# PLK1 in cancer therapy: a comprehensive review of immunomodulatory mechanisms and therapeutic opportunities

**DOI:** 10.3389/fimmu.2025.1602752

**Published:** 2025-06-19

**Authors:** Weihao Wang, Rui Zhao, Yahui Wang, Liying Pan, Fang Luan, Guobin Fu

**Affiliations:** ^1^ Department of Oncology, Shandong Provincial Hospital Affiliated to Shandong First Medical University, Jinan, Shandong, China; ^2^ The Second Clinical Medical College, Shandong University of Traditional Chinese Medicine, Jinan, China; ^3^ Department of Clinical Laboratory, Shandong Provincial Hospital Affiliated to Shandong First Medical University, Jinan, Shandong, China; ^4^ Department of Clinical Laboratory, Shandong Provincial Hospital, Shandong University, Jinan, Shandong, China; ^5^ The Third Affiliated Hospital of Shandong First Medical University, Jinan, China

**Keywords:** cancer, PLK1, inflammation, immune checkpoint inhibitors, cancer vaccines

## Abstract

PLK1 plays a crucial role in cell cycle regulation and cancer development, and its dysregulation has been implicated in the prognosis of a variety of malignancies. The potential of PLK1 inhibitors as cancer therapeutics has been extensively investigated. However, the underlying biology and mechanisms of PLK1 remain incompletely understood. In recent years, numerous studies have demonstrated that PLK1 overexpression is associated with resistance to certain chemotherapeutic agents, while its inhibition can enhance the efficacy of chemotherapy. In addition, PLK1 inhibitors have been shown to selectively target cancer cells as radiation sensitizers and exert synergistic effects in combination immunotherapy. The underlying mechanisms may involve the regulation of multiple immune cells and inflammatory factors, as well as alterations in the tumor microenvironment, ultimately influencing tumor genesis, migration, and invasion. Moreover, PLK1 can regulate the expression of immune checkpoint-related proteins, thereby playing a synergistic role in cancer therapy. Furthermore, PLK1 represents a promising target antigen for cancer immunotherapy, with potential applications in optimizing cancer vaccines. Therefore, this review focuses on the applications and underlying mechanisms of PLK1 in tumor immunotherapy, aiming to provide new insights for improving patient outcomes and prognosis.

## 1 Introduction

PLK1, a member of the polo-like kinase (PLK) subfamily of Ser/Thr protein kinases, plays a pivotal role in regulating diverse cellular processes, including cell cycle progression, differentiation, survival, DNA damage response, autophagy, apoptosis, and cytokine signaling (1, 2). Given its frequent overexpression in various tumor types and its association with poor clinical outcomes, PLK1 has emerged as a highly attractive target for the development of anti-cancer therapies (3). However, due to its critical role in cell cycle regulation, inhibiting PLK1 activity can lead to aberrant mitosis and chromosomal instability in normal tissues. As a result, the use of PLK1 inhibitors carries the risk of inducing new tumors or causing significant side effects in some patients (4).

In recent years, studies have demonstrated that combining PLK1 inhibitors with other therapies can achieve superior efficacy compared to monotherapy. By inhibiting PLK1, these combinations can address the issue of drug resistance to certain chemotherapy agents and act as radiation sensitizers, thereby enhancing the effectiveness of tumor chemoradiotherapy (5–7). Additionally, PLK1 is closely linked to tumor immunotherapy, with its expression showing significant correlations with immunophenotyping, immune cell infiltration, tumor mutational burden (TMB), microsatellite instability (MSI), immune checkpoint gene activity, and therapeutic outcomes across various tumor types (8). In this review, we discuss the structure and function of PLK1, its multifaceted role in cancer biology, and its significance and underlying mechanisms in the context of cancer immunotherapy.

## 2 PLK1 structure and function

In humans, five PLK paralogues have been identified, including PLK1, PLK2 (Snk), PLK3 (Fnk/Prk), PLK4 (Sak), and PLK5 (9). PLK proteins typically comprise two C-terminal Polo-box domains (PBDs) and an N-terminal catalytic kinase domain (10). But PLK4 has three polo box regions, making it the most structurally differentiated member of the PLK family (11). In contrast, PLK5 lacks part of the kinase domain but is still considered a member of the PLK family due to the retention of the PBD sequence (9, 12).

**Figure 1:**
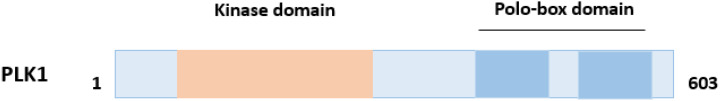
PLK1 protein domains. PLK1 structure includes two functional polo-box domains (PBDs) at C-terminal and the kinase domain at N-terminal.

PLK1 is the most highly conserved member of the polo-like kinase family (**Figure 1**). However, PLK1 gene polymorphisms (such as rs27770, rs40076, rs57973275) may regulate cancer risk and treatment response by affecting its expression, mRNA stability, or function. rs27770 is located in the PLK1 coding region, which can lead to threonine (Thr) to methionine (Met) at position 609. Its allele shows different secondary mRNA structure (13). There are studies have shown that the higher frequency of G allele in Asian population can make PLK1 more susceptible to the inhibition of human microrna: hsa-miR-100-5p, which is more conducive to the prognosis of hepatocellular carcinoma (HCC) (14). rs40076 is located in the intronic region of PLK1, affects mRNA splicing or transcriptional regulation, and can be used as a predictor of bladder cancer susceptibility and survival (15). rs57973275 is located in the 3 ‘-UTR region, and studies have shown that targeting this region can inhibit the expression of PLK1, thereby inhibiting the progression of lung cancer (16).

PLK1 plays a critical role in cell division and is predominantly localized in three distinct subcellular regions: the mitotic centrosomes, kinetochores, and the cytokinetic midbody (17). PLK1 plays a crucial role in centrosome maturation. It also affects the process of centrosome separation in G2/M phase to form bipolar spindle. PLK1 promotes the recruitment of the γ-tubulin ring complex (γ-TuRC) and other Plasma Membrane Calcium-transporting ATPase (PMC) proteins to centrosomes, while phosphorylating key proteins such as the kinase Nek9 and the mitotic motor protein Eg5 (18, 19). On one hand, PLK1 recruits PP2A to BubR1, facilitating the attachment of centromeres to microtubules and maintaining the spindle assembly checkpoint (SAC) (20). On the other hand, once all centromeres are properly attached to spindle microtubules during metaphase, PLK1 is ubiquitinated by the active anaphase-promoting complex/cyclosome (APC/C), leading to its dissociation from centrosomes and the transition out of metaphase (21, 22). Furthermore, PLK1 activity is essential for cytokinesis, as it regulates phosphorylated microtubule-associated protein (PRC1) and intermediate localization protein (CEP55) to mediate cytoplasmic division and abscission (23–25). If PLK1/PRC1 signaling is blocked, tumor growth is inhibited, and drug-resistant tumors become more sensitive to conventional chemotherapy (26).

The process of epithelial cells transdifferentiate into motor mesenchymal cells is called epithelial-mesenchymal transition(EMT). Although EMT is integral in development, wound healing, and stem cell behavior, it also contributes pathologically to fibrosis and cancer progression (27). Studies have shown that PLK1 is a key regulator of EMT in tumor cells. In non-small cell lung cancer, the invasion and metastasis of tumor cells can be promoted by activating the PLK1/β-catenin/AP-1 or PLK1/TGF β axis (28, 29). In addition, PLK1 can accelerate or reverse EMT by regulating the AKT pathway. This phenomenon has been verified in gastric cancer and osteosarcoma (30, 31). In the prostate, PLK1 acts primarily as a potent activator of the MAPK signaling pathway, stimulating cell migration and invasion (32).

## 3 PLK1 and tumors

### 3.1 Effect of PLK1 on tumor development

#### 3.1.1 Tumor-promoting role of PLK1

**Figure 2:**
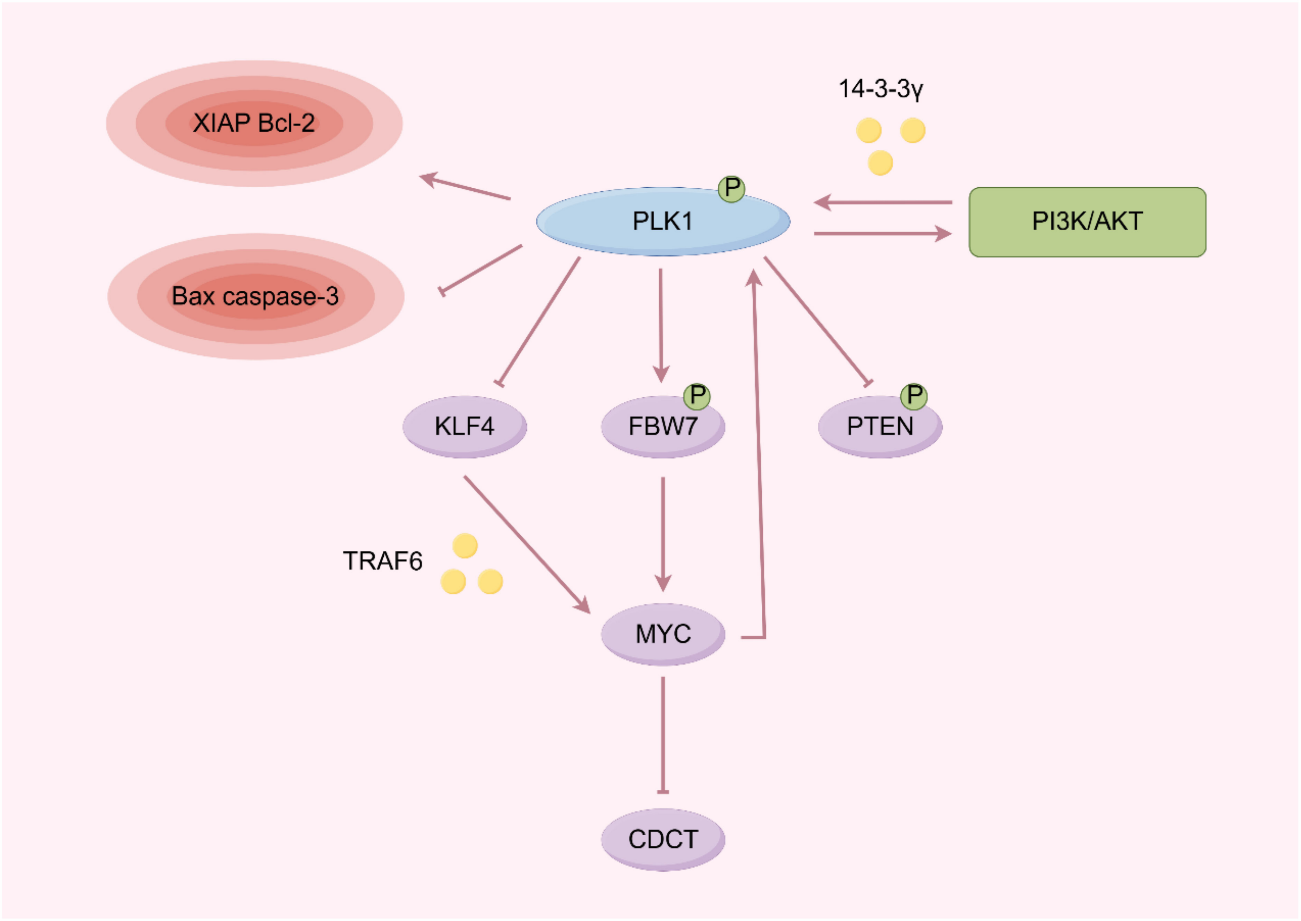
Schematic diagram of the relationship between PLK1 and oncogenes (By Figdraw).

PLK1 is a key regulator of mitosis and cytokinesis, and its overexpression is frequently observed in various tumors, often correlating with poor prognosis (**Figure 2**). PLK1 phosphorylation can inactivate the tumor suppressor gene PTEN, thereby activating the PI3K/AKT pathway, which enhances aerobic glycolysis and promotes tumorigenesis (33). Meanwhile, PTEN is also known to be an important regulator of Plk1 dephosphorylation and chromosome stability during cell division (34). PI3K/AKT could reduce the binding of PLK1 to 14-3-3γ protein, eventually leading to the inability of PLK1 to be activated by catalysis (35). Inhibition of PLK1 can further regulate the downstream genes, including the up-regulation of caspase-3 and Bax and the down-regulation of XIAP and Bcl-2, ultimately affecting the occurrence and development of tumors (36). Furthermore, the combined inhibition of PLK1 and PI3K/AKT, along with FOXO1, exerts synergistic anticancer effects in anaplastic thyroid cancer and non-small cell lung cancer (37, 38). Additionally, PLK1 inhibitors have been shown to enhance the sensitivity of pancreatic cancer to chemotherapy (39).

PLK1 also enhances the stability of the oncogene MYC protein (40, 41). Inhibition of PLK1 reduces the phosphorylation of FBW7, preventing its autoubiquitination and proteasome degradation, thereby promoting the degradation of MYC and reducing tumor cell proliferation while increasing apoptosis (42, 43). PLK1 expression can promote MYC to activate Hedgehog signaling pathway by degrading PDCD4, and then promote the proliferation of tumor cells (44). PLK1 inhibitors also synergistically with mTOR inhibitors to compensatively induce MYC expression, overcome the oxaliplatin resistance of colon cancer, and enhance the radiosensitivity of medulloblastoma (6, 45, 46). At the same time, MYC can deactivate PLK1, preventing PLK1 inhibitors from exerting their effects of sustained activation of SAC and hindering intermediate separation (40, 43, 47, 48).

In nasopharyngeal carcinoma, PLK1 promotes tumorigenesis by mediating KLF4 overexpression. PLK1 directly phosphorylates the Ser234 site of KLF4, leading to the recruitment and binding of the E3 ligase TRAF6, which stabilizes KLF4 through K63-linked ubiquitination. This stabilization enhances the transcriptional activity of KLF4, which in turn activates the MYC oncogenic program, creating a feedforward loop that drives tumor progression (49).

Regulation of the PLK1-p53 signaling axis eventually induces cell cycle arrest and inhibits tumor growth (50). P53 can promote the cytotoxicity of PLK1-targeted therapy and reduce tumor recurrence and metastasis (51). Other transcription factors regulated by PLK1 include PLK1 phosphorylation-dependent REST degradation in triple-negative breast cancer (52), SUZ12 and ZNF198 in hepatitis B virus (HBV)-mediated liver cancer (53), and transcription of the key tumor suppressor FOXO1 in prostate cancer cells and rhabdomyosarcoma (54, 55). PLK1 can also exert a synergistic effect with FOXM1 to affect the prognosis of liver cancer (56), papillary thyroid cancer (57), bladder cancer (58),diffuse large B-cell lymphoma (59), kidney cancer (60), lung cancer (61), breast cancer (62), esophageal cancer (63) and other tumors.

In addition, PLK1 can also coordinate cancer progression by influencing metabolic reprogramming. PLK1 plays a key role in the biosynthesis of cancer cells by promoting the formation of glucose-6-phosphate dehydrogenase (G6PD) active dimers, interacting with them and directly phosphorylation, thereby activating the pentose phosphate pathway (PPP) (64). PLK1 is also a valuable molecular target for angiogenesis, and inhibition of its expression can inhibit the formation of new tubular structures in non-small cell lung cancer and prostate cancer, and enhance the chemotherapy sensitization of paclitaxel in cancer cells (65) (**Table 1**). Recent studies have also found that PLK1 is involved in ferroptosis pathway, and PLK1-CBx8-GPX4 can overcome the drug resistance mechanism of colorectal cancer by inducing ferroptosis (66, 67).

**Table 1:** Overview of PLK1 substrates and functions related to cancer.

Substrate/Pathway activation	P-Site
Inhibition of tumor suppressors	
PTEN	Ser385
REST	Ser1030
SUZ12	Ser539,546
ZNF198	Ser305
FOXO1	Ser75
Activation of tumor promotors	
FBW7	Ser58/Thr284
KLF4	Ser234
Metabolism	
G6PD	Thr406,466

#### 3.1.2 PLK1 as a tumor suppressor

Despite its predominant role as a tumor promoter, PLK1 can also exhibit tumor-suppressive effects under certain conditions. For instance, PLK1 overexpression has been shown to inhibit the development of Kras- or Her2-induced breast tumors by interfering with mitotic processes and cytokinesis (68). Additionally, high levels of PLK1 have been associated with improved survival rates in colorectal cancer patients with APC mutations. In these cases, inhibition of PLK1 in colon cells expressing mutant APC-ΔC disrupts spindle assembly checkpoint (SAC) recruitment by reducing the localization of BUBR1 and MAD1 at the centromere, leading to chromosomal abnormalities and an increased number of intestinal tumors in APC Min/+ mice (69).

### 3.2 PLK1 as a target for cancer therapy

Given its central role in cell cycle regulation and its elevated expression in various cancers, PLK1 has emerged as a promising target for cancer therapy. Inhibition of PLK1 has been shown to enhance the sensitivity of tumors to chemotherapy and radiotherapy (70). PLK1 inhibitors can be broadly categorized into ATP-competitive inhibitors that target the kinase domain (KD) and compounds that target the polo-box domain (PBD) (71).

BI 2536 is a potent ATP-competitive inhibitor that inhibits tumor growth *in vivo* and *in vitro (* 72). Clinical trials have shown that BI 2536 can exert synergistic anti-tumor effects when combined with other chemotherapeutic agents, but the efficacy of single therapy is poor (73). Volasertib, an ATP-competitive inhibitor, exhibits sustained high-dose exposure in tumor tissues and has demonstrated antitumor activity and a favorable safety profile in multiple xenograft models (74). Overexpression of the ATP-binding cassette (ABC) drug transporter ABCB1 has been shown to cause ATP hydrolysis, which contributes to drug resistance in Volasertib. Therefore, combination therapy with ABCB1 modulators can be considered as a way to solve the problem of drug resistance (75). In addition, Volasertib exerts synergistic effects when combined with both MEK inhibitors and histone deacetylase (HDAC) inhibitors (76, 77). Onvansertib is a novel ATP-competitive PLK1 specific inhibitor, which can induce mitotic cycle arrest and apoptosis in tumor cells. Thus, the growth of xenograft tumors was inhibited (78). It is currently being tested in three clinical trials in combination with standard therapy (KRAS-mutated metastatic colorectal cancer, acute myeloid leukemia, and castration-resistant prostate cancer) and has shown promising drug resistance and safety profiles (79–81). However, this ATP-competitive PLK1 inhibitor has certain limitations. Firstly, the kinase domains of PLK family members (such as PLK1/2/3) are highly similar, resulting in reduced selectivity of existing drugs and easy to cause off-target effects. Secondly, PLK1 has complex and pleiotropic functions in the cell cycle regulatory network. When the inhibitor concentration is too high, PLK2/3 and other paraloproteins are unspecifically inhibited, resulting in dose-dependent cytotoxicity (such as bone marrow suppression and gastrointestinal reactions) (4). Finally, acquired mutations in the ATP-binding domain in tumor cells, such as Gatekeeper mutations in the kinase domain, may lead to decreased drug-binding affinity for PLK1 inhibitors and thus the phenomenon of acquired resistance (82).

Non-ATPcompetitive inhibitors, such as Rigosertib, which targets the PBD and inhibits both PLK1 and PI3K, have shown efficacy in killing tumor cells *in vitro* and *in vivo*. PBD inhibitors are more specific because of their high affinity for specific residues of PLK1 compared to ATP competitive inhibitors (71). Poloxin, a PBD inhibitor developed in 2008, causes mitotic arrest and apoptosis of cancer cells by inducing centrosome fragmentation and abnormal arrangement of spindles and chromosomes. In the xenograft mouse model, Poloxin was also shown to inhibit tumor growth. However, Poloxin is easily degraded *in vitro* and *in vivo*, resulting in a short half-life and difficult to maintain its efficacy. Moreover, due to its large molecular polarity and poor transmembrane permeability, it is difficult to reach an effective concentration in tumor cells (83, 84). However, Rigosertib has underperformed in clinical trials for high-risk myelodysplastic syndromes and metastatic pancreatic cancer, with patients showing no significant benefits over standard care. On the one hand, the phosphopeptide binding interface of PBD is relatively shallow. On the other hand, it may activate other mitotic kinases (such as Aurora A/B, WEE1) to compensate for PLK1 function (71, 85). However, some studies have shown that Rigosertib can also target the RAS pathway and overcome chemotherapy resistance. Therefore, combining this inhibitor with chemotherapy may improve efficacy in patients with KRAS mutations (86). Therefore, further research is needed to improve the specificity and reduce the resistance of PLK1 inhibitors, making them more effective in clinical applications. Recent studies have found that dual-target inhibitors targeting PLK1/BRD4 and PLK1/MEK can produce cumulative effects and exert long-term inhibitory effect on cancer cell growth (87–89) (**Table 2**).

**Table 2:** PLK1 inhibitors in clinical trials.

Compound	Target	Clinical Trial Phase	Indication	NCT number
BI 2536	ATP- binding	I	Advanced solid tumors	00526149
		II	Small cell lung cancer	00412880
		II	AML/MDS	00422890
Volasertib	ATP- binding	III	AML	01721876
Onvansertib	ATP- binding	II	Metastatic colorectal cancer with a KRAS mutation	03829410
		II	Metastatic castration-resistant prostate cancer	03414034
		II	Metastatic pancreatic ductal adenocarcinoma	04752696
Poloxin	Polo-box domain			
Rigosertib	Polo-box domain	III	Untreated metastatic pancreatic cancer	01360853
		III	Myelodysplastic syndrome	01928537
		III	Myelodysplastic syndrome	02562443

## 4 PLK1 and immunity

**Figure 3:**
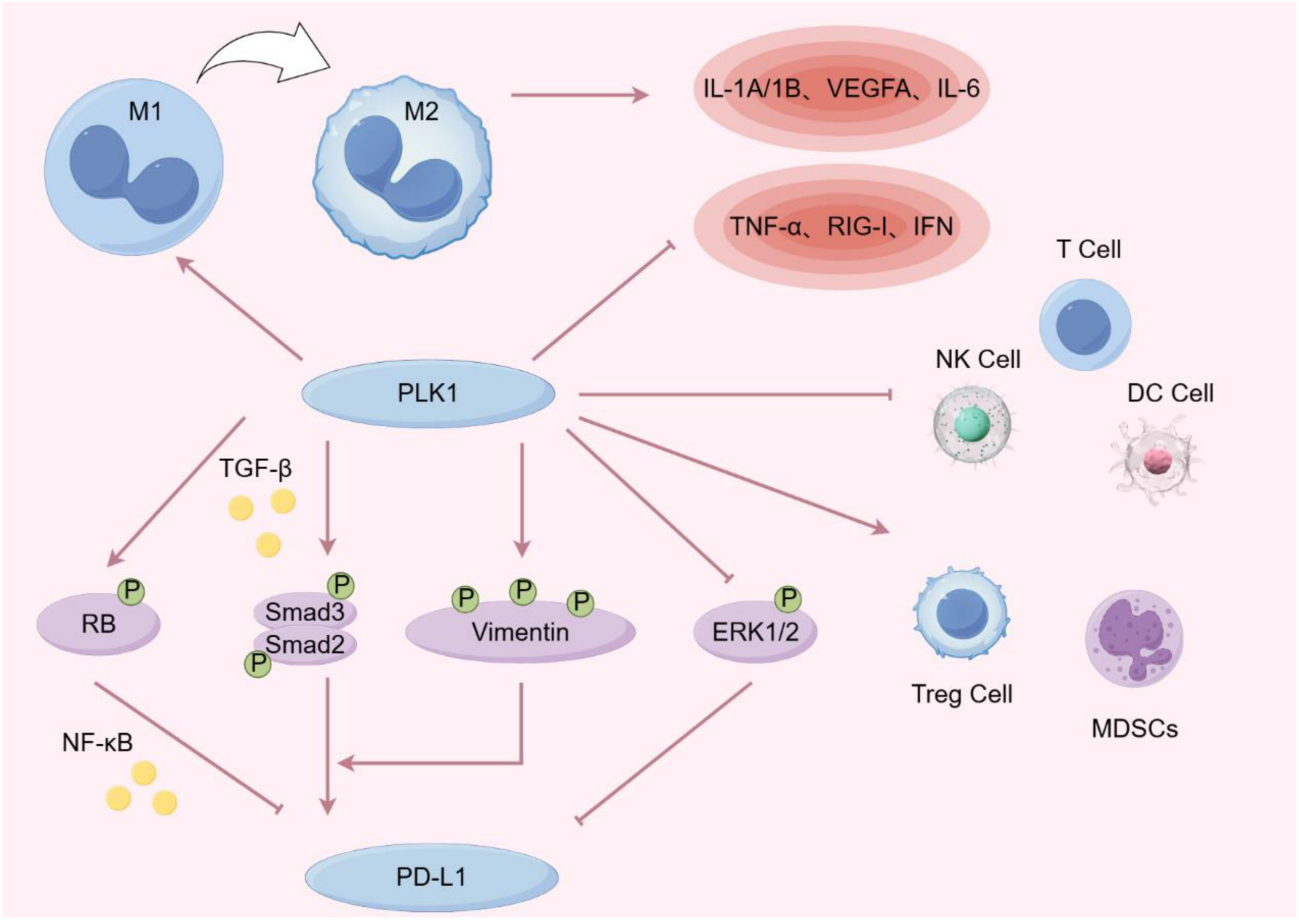
PLK1 decreased the levels of TNF-α, RIG-1 and IFN, promoted the polarization of tumor-associated macrophages from M1 to M2, and increased the levels of IL-1A, VEGFA and IL-6. PLK1 was negatively correlated with DC cells, T cells and NK cells, and positively correlated with myeloid suppressor cells (MDSCs) and regulatory T cells. PLK1 affects PD-L1 expression by regulating MAPK, TGF-β and NF-ĸB signal transduction pathways. (By Figdraw).

PLK1 has been found to play a significant immunomodulatory role in almost all types of cancer. The following pathways are mainly involved: immune cell infiltration, inflammatory signaling, immune checkpoint inhibitors and cancer vaccines. Increased PLK1 expression can inhibit the function of immune cells, such as NK cells and T cells, thereby promoting tumor immune escape (90). PLK1 also regulates inflammatory mediators and cellular effectors, thereby altering the local tumor microenvironment to promote tumor cell proliferation and survival while disrupting the adaptive immune response (91). Additionally, PLK1 exhibits positive associations with multiple immune checkpoints, encompassing both immunosuppressive and immunostimulatory checkpoints (92, 93). Moreover, PLK1 represents an attractive target antigen for cancer immunotherapy, playing a crucial role in the optimization of cancer vaccines (94) **Figure 3**).

### 4.1 PLK1 and immune cells infiltration

The tumor microenvironment (TME) is a critical determinant of cancer cell survival and metastasis, and the infiltration of immune cells into the tumor is a key factor influencing the efficacy of immunotherapy (95, 96). Accumulating evidence indicates that PLK1, beyond its well-established role in mitosis, exerts significant effects on the tumor microenvironment and is involved in tumor cell metastasis and immune cell infiltration (8, 97).

On one hand, PLK1 regulates the infiltration of a variety of immune cells. In lung cancer, PLK1 inhibits DC maturation and T-cell enrichment (98, 99).Moreover, PLK1 expression is positively correlated with myeloid-derived suppressor cells (MDSCs) and regulatory T cells. In addition, PLK1 was positively correlated with myeloid-derived suppressor cells (MDSCs) and regulatory T cells in breast cancer and node-predominant Hodgkin’s lymphoma (100–102). In summary, PLK1 modulates the function of immune cell infiltration, providing a foundation for the development of inhibitors targeting innate immune maintenance.

On the other hand, PLK1 has been shown to promote the polarization of tumor-associated macrophages (TAMs) from the M1 to the M2 phenotype (103). Tumor-associated macrophages can be polarized into two distinct phenotypes: M1 macrophages, which activate T-helper type 1 (Th1) T cells to induce a cytotoxic T-cell response against pathogens, thereby exerting tumor-killing activity and increasing hypoxia; and M2 macrophages, which promote tissue repair and wound healing, participate in angiogenesis, and secrete a variety of pro-inflammatory factors with pro-tumorigenic effects, thereby contributing to cancer progression (104, 105). In hepatocellular carcinoma, PLK1 interacts with PTEN and interferes with its nuclear translocation, leading to the inhibition of natural killer (NK) cell and T cell function by enhancing aerobic glycolysis and promoting M2 macrophage polarization (106). High expression of PLK1 inhibits the infiltration of M1 macrophages and their associated chemokines and marker genes into the glioblastoma immune microenvironment, whereas knockdown of PLK1 increases the infiltration and polarization of M1 macrophages (107).

In addition, PLK1 inhibitors can prevent and treat acute graft-versus-host disease (aGvHD) that may occur after leukemia transplantation by preventing activation and inducing apoptosis of already activated alloreactive T cells (Tallo cells) while inhibiting the molecular chaperone Hsp90 and inhibiting Tallo cell proliferation (108). There is also a link between PLK1 and Toll-like receptor (TLR) signaling, which plays a key role in innate immunity. TLRs are important sentinels of bacterial and viral infections, and PLK inhibitor-mediated blockade of TLR signaling can lead to adverse effects. Thus, in some patients receiving PLK1 inhibitors during cancer treatment, the risk of infection with invading microorganisms may be increased due to the impaired ability of the TLR recognition system to sense and initiate a cytokine response (109).

### 4.2 PLK1 and inflammatory factor

Inflammatory factors are a wide range of effector molecules involved in inflammatory response, including a variety of cytokines and chemokines. Many cancers arise from sites of infection, chronic irritation, and inflammation. Studies have shown that inflammatory factors can act directly on tumor cells. Inflammatory cells also modulate tumor growth by orchestrating the tumor microenvironment and influencing the adaptive immune response. Exploring the role of inflammatory factors in tumor immunity can provide a new way for cancer treatment in the future (110, 111).

An earlier study in 2013 found that PLK1 inhibitors could help treat colon cancer or early-stage lesions with high levels of inflammatory cell infiltration (112). Some studies have suggested that the high selectivity of PLK1 inhibitors for the BET bromodomain is the reason why PLK1 inhibitors can be used as drug targets for cancer and inflammation (113). Further studies have found that the activation of PLK1 inhibits the expression of TNF-induced cyclin D1, providing a potential mechanism for TNF-α’s involvement in inflammation-induced cancer (114). Recently, PLK1 has been identified as a novel negative regulator of the RIG-I and IFN-inducing pathways. RIG-I is capable of upregulating the expression of pro-inflammatory cytokines, inducing inflammation in the tumor environment, and activating neighboring immune cells, while IFN plays an important role in T cell proliferation, antigen sensitivity, cytokine production, and migration (115, 116). PLK1 also promotes the polarization of tumor-associated macrophages to upregulate the expression of IL-1A/1B, VEGFA, and IL-6, and the increased activity of these genes and factors is inversely associated with survival in patients with advanced lung adenocarcinoma (61).

Studies have highlighted the role of PLK1 in regulating inflammatory factors in sepsis and related diseases, offering potential insights for anti-inflammatory strategies in tumor treatment. Sepsis and cancer share several pathophysiological features, and the immune dysfunction associated with sepsis may influence the progression of malignant tumors (117, 118). For instance, in sepsis-induced acute lung injury (ALI), the activation of the ROS-mediated NLRP3 inflammasome is suppressed through modulation of the PLK1/AMPK/DRP1 signaling axis (119). Additionally, PLK1 has been identified as a key contributor to sepsis-induced myocardial dysfunction (SIMD). Its expression is upregulated in lipopolysaccharide (LPS)-treated mouse hearts and neonatal rat cardiomyocytes (NRCM). Inhibition of PLK1 attenuates the activation of the NF-κB signaling pathway, thereby mitigating LPS-induced myocardial injury, inflammation, and cardiac dysfunction (120). In contrast, PLK1 exhibits a protective role in sepsis-induced intestinal barrier dysfunction. Overexpression of PLK1 reduces IL-6 levels by suppressing NF-κB signaling, thereby alleviating intestinal epithelial damage (121, 122).

### 4.3 PLK1 and immune checkpoint inhibitors

Programmed death-ligand 1 (PD-L1) is widely expressed in human tumors and plays a critical role in immune evasion. By binding to its receptor PD-1 on activated T cells, PD-L1 inhibits T cell activation signaling, thereby promoting tumor immune escape (123). Blockade of the PD-L1/PD-1 pathway has shown significant anti-tumor effects in patients with advanced cancer and is considered the gold standard for developing new immune checkpoint blockade (ICB) therapies and combination treatments. However, the response rate to anti-PD-L1 antibodies remains limited in several solid tumors (124). Consequently, improving the sensitivity of cancer patients to immune checkpoint inhibitors and expanding the population benefiting from immunotherapy are critical areas of future research.

Recent studies have revealed that PLK1 inhibitors can synergize with PD-L1 immune checkpoint inhibitors in tumor immunotherapy. Analysis of the TCGA dataset identified PLK1 as one of the proliferation-related kinases highly expressed in cancers with chromosome 9p copy number gains (CNGs) involving PD-L1. This finding is relevant to a variety of cancers, including lung cancer, melanoma, bladder cancer, head and neck cancer, cervical cancer, soft tissue sarcoma, prostate cancer, gastric cancer, ovarian cancer, and triple-negative breast cancer (125).

Research has shown that PLK1-induced phosphorylation of Vimentin promotes PD-L1 expression by activating TGF-β signaling and interacting with p-Smad2/3, contributing to metastatic progression in lung adenocarcinoma (LUAD) (126). Meanwhile, *in vivo* experiments confirmed that PLK1 inhibitor combined with PD-L1 inhibitor could significantly reduce tumor progression in mice compared with the two drugs alone. Because inhibition of PLK1 can induce the up-regulation of PD-L1 through the MAPK pathway and enhance the sensitivity of tumor cells to immune checkpoint inhibitors (127). Additionally, there are studies reported that PLK1 inhibition suppresses Rb phosphorylation in lung cancer. In various cancer cell lines, phosphorylated Rb inhibits the transcriptional activity of NF-κB and the expression of PD-L1 mRNA (128, 129). These findings suggest that PLK1 inhibitors may influence tumor immunotherapy efficacy in lung cancer by modulating the Rb/NF-κB/PD-L1 axis.

In pancreatic cancer, PLK1 inhibition similarly upregulates PD-L1 expression but also enhances sensitivity to PD-L1 blockade, ultimately leading to tumor suppression. This combination therapy can transform immunologically “cold” tumors into “hot” tumors, thereby improving anti-tumor immune responses. Mechanistically, PLK1 inhibition or depletion increases nuclear localization of NF-κB by reducing Rb phosphorylation, which upregulates PD-L1 expression (130). This mechanism supports the proposed role of the PLK1/Rb/NF-κB/PD-L1 axis in lung cancer and highlights its potential relevance across multiple cancer types.

The article published reported that PLK1 has the ability to alter the transcriptional profile of Her2+ breast tumors in the living environment, affecting the effector capacity of NK and T cells. This coordinated interaction in the tumor microenvironment ultimately upregulates PD-L1 and CD206 in the later stages of tumor progression and induces the NF-kb signaling pathway, promoting immune evasion (131).

### 4.4 PLK1 and cancer vaccines

Genetic instability in tumor cells often results in a high frequency of mutations, and the expression of non-synonymous mutations can generate tumor-specific antigens known as neoantigens. These neoantigens are highly immunogenic because they are not expressed in normal tissues. Neoantigen-targeting cancer vaccines primarily include nucleic acid vaccines, dendritic cell vaccines, tumor cell vaccines, and synthetic long-peptide vaccines (SLPs) (132).

In recent years, it has been found that PLK1 may be a universal tumor antigen recognized by cytotoxic T lymphocytes for cancer immunotherapy. PLK1-specific CD4(+) and CD8(+) T cells can be induced by mPLK1 RNA/DC vaccine and exert anti-tumor effects (94). Inoculation of bone marrow cells CD8 T against the cellular epitope synthesis of PLK1: PLK1122 (DSDFVFVVL) yields a large number of long-lasting antigen-specific CD8 T cells. The use of a peptide vaccine that simultaneously targets PLK1 and blocks PD-L1 can lead to complete tumor eradication and long-term survival in mice with clonal heterologous C1498 myeloid leukemia (133).

However, PLK1 as a target of direct immunotherapy still faces multiple challenges, mainly involving target specificity, immunogenicity and tumor heterogeneity. PLK1 still has basal expression in normal proliferating cells. Systemic targeting of PLK1 may cause “on-target, off-tumor” toxicity, leading to bone marrow suppression or gastrointestinal injury. Such risks may be further amplified by the long-term activating effects of immunotherapy (134). PLK1 did not show strong immunogenicity in human models, and experimental data showed that PlK1-derived peptides only weakly activated T cells in peripheral blood of patients (135). In addition, in a study utilizing a DNA vaccine model to compare the immunogenicity of G2/M-related antigens, it was observed that PLK1-immunized mice did not exhibit any anti-tumor effects. This may be related to the fact that PLK1 is not preferentially expressed in the cancer stem cell (CSC) or cancer initiation cell (CIC) population (136). In summary, PLK1 may be an attractive target antigen for cancer immunotherapy, but is more likely to act as an accessory target rather than an independent immunotherapy antigen.

## 5 Conclusions and future perspectives

PLK1 is a key regulator of mitosis, playing critical roles in centrosome maturation and separation, spindle assembly formation, and cytokinesis. Due to its central role in cell division, aberrant expression of PLK1 can have profound detrimental effects, particularly in carcinogenesis. This review focuses on the relationship between PLK1 and cancer development. On one hand, PLK1 promotes tumor progression through the PLK1/β-catenin/AP-1 axis or the PLK1/TGF-β axis, while on the other hand, it activates the AKT and MAPK pathways, leading to epithelial-mesenchymal transition (EMT) and ultimately facilitating tumor cell invasion and metastasis. Additionally, PLK1 is widely recognized as a cancer-promoting gene that regulates multiple tumor suppressor gene inactivation and proto-oncogene expression. Inhibition of PLK1 has been shown to enhance chemosensitivity, highlighting its potential as a therapeutic target.

PLK1 also modulates the expression of transcription factors across various cancer types, further contributing to its tumor-promoting functions. However, despite its oncogenic role, only a limited number of PLK1 inhibitors have demonstrated promising therapeutic effects in clinical trials. This is largely due to challenges such as toxicity at high doses, the pleiotropic functions of PLK1 in mitotic cells, and off-target effects. Interestingly, PLK1 overexpression has been found to inhibit tumor development in certain contexts by disrupting mitotic progression, spindle assembly checkpoint recruitment, and cytokinesis. These dual roles suggest that PLK1 acts as a double-edged sword, capable of either promoting or suppressing tumor development depending on the context. This complexity complicates the therapeutic application of PLK1 inhibitors and underscores the need for further research to fully understand its functions.

In recent years, increasing evidence has highlighted the significant role of PLK1 in cancer immunotherapy. Firstly, PLK1 plays a crucial role in cancer progression by modulating the tumor microenvironment, particularly through the regulation of immune cell infiltration and inflammatory factors. PLK1 inhibits the recruitment of immune-promoting cells such as dendritic cells (DCs), T cells, and NK cells, while positively correlating with the presence of myeloid-derived suppressor cells (MDSCs) and regulatory T cells. Additionally, PLK1 acts as a negative regulator of key inflammatory mediators, including TNF-α, RIG-1, and IFN, and is implicated in inflammation-driven cancer processes. Furthermore, PLK1 promotes the polarization of tumor-associated macrophages (TAMs) from the M1 to the M2 phenotype, upregulating the expression of chemokines such as IL-1A/1B, VEGFA, and IL-6, which are associated with poor survival outcomes in cancer patients.

Secondly, PLK1 inhibitors have shown synergistic effects when combined with PD-L1 immune checkpoint inhibitors in tumor immunotherapy. These effects are primarily mediated through the TGF-β, MAPK, and NF-κB signaling pathways, enhancing the anti-tumor immune response. Lastly, PLK1 has emerged as a universal tumor antigen recognized by cytotoxic T lymphocytes, making it a promising target for cancer immunotherapy. Dendritic cell vaccines targeting PLK1-specific CD4(+) and CD8(+) T cells have demonstrated potent anti-tumor effects. Similarly, peptide vaccines derived from PLK1 epitopes have been shown to generate long-lasting antigen-specific CD8 T cells, leading to complete tumor eradication and prolonged survival in murine models. Notably, a polypeptide vaccine targeting both PLK1 and PD-L1 has shown remarkable efficacy in eliminating tumors and improving survival outcomes in mice.

In conclusion, expanding our understanding of PLK1 signaling in immunotherapy offers new avenues to enhance the efficacy of PLK1 inhibitors and improve the sensitivity of immunotherapeutic approaches. These insights also provide a foundation for the development of novel therapeutic strategies, paving the way for more effective cancer treatments.

## Glossary

**Table:**

Abbreviations	Full English name	Definition
TMB	Tumor Mutational Burden	Measure of mutations carried by tumor cells
MSI	Microsatellite Instability	Condition of genetic hypermutability due to impaired DNA mismatch repair
PBD	Polo-Box Domain	Protein interaction domain of PLK family kinases
PMC	Perivascular Macrophage Cluster	Specialized macrophage subset around blood vessels
PRC1	Protein Regulator of Cytokinesis 1	Key regulator of mitotic spindle formation
CEP55	Centrosomal Protein 55	Essential component of the centrosome
EMT	Epithelial-Mesenchymal Transition	Cellular reprogramming process in metastasis
G6PD	Glucose-6-Phosphate Dehydrogenase	Rate-limiting enzyme in pentose phosphate pathway
SAC	Spindle Assembly Checkpoint	Critical mitotic quality control mechanism
KD	Kinase Domain	Catalytic domain of protein kinases
HDAC	Histone Deacetylase	Enzyme modifying chromatin structure
TME	Tumor Microenvironment	Cellular environment surrounding tumors
DC	Dendritic Cells	Professional antigen-presenting cells
MDSCs	Myeloid-Derived Suppressor Cells	Immunosuppressive myeloid population
TAMs	Tumor-Associated Macrophages	Pro-tumor macrophages in TME
aGvHD	acute Graft-versus-Host Disease	Donor immune cell attack on recipient
TCR	T Cell Receptor	Antigen recognition complex of T cells
ACI	Adoptive Cell Immunotherapy	Therapeutic T cell transfer
SIMD	Spindle and Midbody Database	Repository of mitotic proteins
NRCM	Neonatal Rat Cardiomyocytes	Common cardiac cell model
ICB	Immune Checkpoint Blockade	Therapy targeting PD-1/CTLA-4 etc.
CNGs	Cancer Neoantigens	Tumor-specific mutated antigens
LUAD	Lung Adenocarcinoma	Major NSCLC subtype
SLPs	Synthetic Lethal Pairs	Gene pairs where dual loss is lethal
CSC	Cancer Stem Cells	Tumor-initiating cell population
CIC	Cancer Initiating Cells	Alternative term for CSCs
